# Spinal muscular atrophy in Ghanaian children confirmed by molecular genetic testing: a case series

**DOI:** 10.11604/pamj.2023.46.78.32240

**Published:** 2023-11-09

**Authors:** Charles Kumi Hammond, Emmanuel Oppong, Emmanuel Ameyaw, Joslin Alexei Dogbe

**Affiliations:** 1 Department of Child Health, Kwame Nkrumah University of Science and Technology, Kumasi, Ghana,; 2 Department of Child Health, Komfo Anokye Teaching Hospital, Kumasi, Ghana

**Keywords:** Spinal muscular atrophy, motor neuron disease, floppy infant, survival motor neuron

## Abstract

Spinal muscular atrophy (SMA) is an autosomal recessive inherited motor neuron disease characterized by progressive muscle weakness due to degeneration and loss of the anterior horn cells in the spinal cord and the brain stem nuclei from foetal life through infancy and childhood. SMA is prevalent in Ghanaian children, though not widely reported. Cases are likely missed or misdiagnosed due to lack of expertise and investigations. Newborn screening is not currently available in Ghana. The management remains supportive as newly approved genetic modifications therapies are currently not available. We present a retrospective folder review of children attending a tertiary pediatric neurology clinic who were diagnosed with SMA and confirmed by molecular genetic testing. Between January 2018 and August 2021, five (5) children from three families had molecular genetic tests confirming their diagnosis of SMA. Three (3) children had SMA I phenotype while 2 had SMA III phenotype. Two (2) of the 3 children with SMA I died from respiratory complications. The last surviving child with SMA I was diagnosed through newborn screening program overseas and received gene modification therapy. Careful history and physical examination remain the best approach to diagnosis as confirmatory genetic testing and supplemental investigations are not readily available. The current management of the children with SMA in Ghana include respiratory care, physiotherapy, and genetic counselling. Genetic modification therapies are currently not available.

## Introduction

Spinal muscular atrophy (SMA) is an autosomal recessive inherited motor neuron disease characterized by progressive muscle weakness due to degeneration and loss of the anterior horn cells in the spinal cord and the brain stem nuclei from foetal life through infancy and childhood [1]. In 94% of affected individuals, there is a homozygous deletion in the *Survival Motor Neuron* 1 (SMN1) gene located on chromosome 5q11.2-13.3 [2]. Spinal muscular atrophy is the most common genetic cause of infant mortality [3,4] with an estimated global incidence of 1 in 6,000-10,000 live births and a carrier frequency of 1 in 47-72 individuals [1,5]. The phenotypic expressions of SMA spans a broad continuum and is determined by the copy numbers of the *Survival Motor Neuron* 2 (SMN2). Spinal muscular atrophy is clinically classified based on the age at onset and the peak motor function into a severe infantile form (SMA type I or Werdnig-Hoffmann disease), a late infantile type (SMA type II or intermediate type) and a mild or juvenile form (SMA type III or Kugelberg-Welander disease). In SMA type I, the onset is <6 months of age. In type II, the onset is between age 6-18 months, and in type III after age 18 months. A severe foetal form (SMA type 0) is usually fatal and is associated with arthrogryposis multiplex congenita. Spinal muscular atrophy type IV, the mildest form, is seen in adults [1]. Spinal muscular atrophy I is the commonest form of SMA. Affected infants may appear normal at birth, but before age 6 months present with floppiness, symmetric generalized muscle weakness which is more proximal than distal, and absent deep tendon reflexes. Patients usually have an alert facial expression with preserved cerebral function. However, weakness of the bulbar muscles results in poor swallow reflexes, pooling of secretions, tongue fasciculations, and an increased risk of aspiration and respiratory infections. There is intact sensation [1]. Affected infants can have paradoxical breathing due to selective involvement of the intercostal muscles but not the diaphragm. The loss of axial tone shows as excessive head lag when pulling the infant to sit and an “inverted U” posture on horizontal suspension. They do not attain independent sitting [1].

Most infants with SMA I die from respiratory failure from progressive muscle weakness and respiratory tract infections within the first year of life. With multidisciplinary care that offers optimum respiratory and nutritional support, patients may survive up to their second birthday [6]. The intermediate form (SMA II) has a more slowly progressive course. The affected children can sit unsupported but do not attain independent walking. They can live into late childhood. The mild or juvenile form (SMA III) presents with muscle weakness after age 18 months [1]. These children are able to walk independently at some point in life and can survive into adulthood. Confirmation of the diagnosis of SMA is by molecular genetic testing with targeted mutation analysis which can detect homozygous deletions of exons 7 and 8 of SMN1 gene [4]. Other useful investigations include neurophysiological tests, muscle biopsy and electrocardiography. Motor nerve conduction studies will typically demonstrate diminished amplitude while sensory nerve conduction studies are normal. Electromyogram may show non-specific findings such as positive sharp waves and fibrillation. The electrocardiogram (ECG) typically demonstrates a baseline tremor as an artefact representing muscle fibrillations. Muscle biopsy shows group atrophy in types I and II muscle fibres, indicating muscle denervation [1,4]. The management of children with SMA in limited-resource settings is mainly supportive and includes respiratory care such as non-invasive respiratory support or tracheostomy and frequent suctioning of oral secretions during intercurrent illness. Patients in steady states should be offered regular assessment of respiratory function. Nocturnal positive-pressure breathing prevents daytime fatigue caused by sleep apnoea [1]. Proper seating and orthosis delay the development of scoliosis and allow the patient to be upright rather than bedridden. The use of electric wheelchairs improves mobility and allows the children to be integrated into mainstream schools [1]. Recently, genetic therapies have been developed to treat children with SMA. They modify the genes for the SMN protein and have shown promise in improving the phenotypes of children with SMA [7,8]. These are currently not available in resource-limited settings. A literature search for cases of SMA in Ghana revealed 6 cases reported between 1992 and 2016 [9,10]. None of these cases, however, was confirmed by molecular genetic testing due to its non-availability within the public health sector. We report five additional cases of SMA in Ghanaian children from three different families all of which were confirmed by genetic testing.

## Methods

The Komfo Anokye Teaching Hospital is the second largest tertiary hospital in Ghana. The Pediatric Neurology Unit runs a twice-weekly child neurology out-patient clinic which receives referrals from the middle and northern belts of the country. There are over 2000 visits to the clinic annually. A search through the clinic database for “spinal muscular atrophy” or “floppy infant” showed that there were 13 cases of suspected SMA seen at the clinic from January 2018 to August 2021. Of these, 5 had genetic confirmation of SMA. All the genetic tests were done in private laboratories where the samples were sent overseas to be tested. It took an average of 2 weeks to receive the test results. We embarked on a retrospective folder review of these cases. Written informed consents were obtained from parents of these children for their details to be included in this study.

## Results

The demography, clinical presentations, management, and long-term outcomes of the 5 cases of SMA confirmed by molecular genetic testing are presented below. Table 1 summarizes the details of these cases. In addition, we present the family pedigree of two siblings showing the detailed description of the genotypes of their immediate family members.

**Table 1:** summary of cases of SMA confirmed by molecular genetic testing

Cases	Year presented	Age/sex	Clinical presentation	Molecular genetic testing	SMA phenotype	Management & outcome
1	2018	4mo, male	Floppy infant, severe head lag, delayed motor milestones, tongue fasciculations, absent DTR; admitted to hospital 2x for chest infection	Homozygous deletion of SMN1 gene; SMN2 copy numbers not determined	SMS I	Died aged 9mo in a district hospital from a chest infection
2	2019	3mo, female	Floppiness and frequent chocking during feeds; decreased muscle tone and power, absent reflexes; had paradoxical breathing	Homozygous deletion of SMN1 gene; SMN2 copy numbers not determined	SMA I	Died at age 7mo. after aspiration during feeding
3	2018	8y, female	Delayed motor milestones; gained independent walking briefly at age 3 years and regressed a few weeks afterwards; had muscle weakness (proximal > distal), absent DTR, and thoracolumbar scoliosis; has advanced speech and social developments	Homozygous deletion of SMN1 gene (exons 7&8); SMN2 copy numbers not determined	SMA III	Doing well at 11y of age; attends physiotherapy; transitioned into an electric wheelchair; Her home has been redesigned to be disability friendly; her school is keeping her class on ground floor
4	2020	4y, male	Delayed motor milestones; walked independently at 18mo; still ambulating at 4y with a waddling gait and lordotic stance;has Gower’s sign	Homozygous recessive for SMN1 mutation (exon 7&8);heterozygous duplication of SMN2 (exons 7&8); 3 copy numbers of SMN2 gene	SMA III	Doing well at 4y; still ambulating; attends mainstream education
5	2021	9mo, female	Born in the US and diagnosed SMA through newborn screening; presented with generalized hypotonia, muscle weakness and absent DTR	SMN1 mutation identified through newborn screening	SMA I	Received gene therapy at age 4mo; currently 18mo old. Has been admitted once for chest infection plans to start nocturnal BiPAP ventilation at home and to insert gastrotomy tube for feeding

BiPA: bivalve invasive positive airway pressure; DTR: deep tendon reflexes; SMA: spinal muscular atrophy; SMN: survival motor neuron; mo: months; y: years

**Figure 1:**
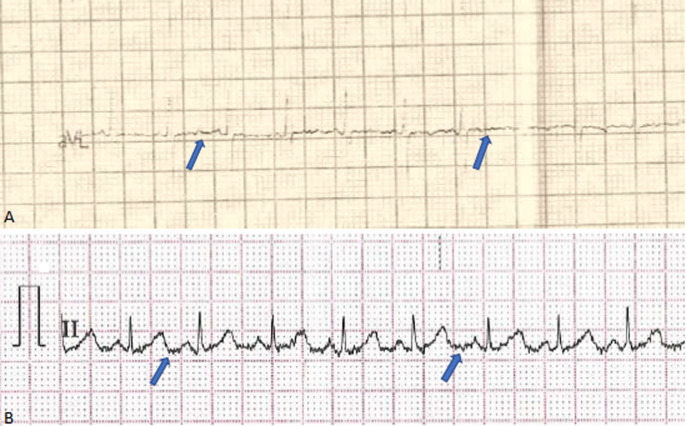
A,B) electrocardiogram for case 1 and case 5 showing baseline fibrillations

**Case 1:** a 4-month-old male infant born to non-consanguineous Ghanaian parents with no known family history of neuromuscular disorders, was referred to the paediatric neurology clinic in 2018 with generalised hypotonia and delayed motor milestones. His mother perceived adequate foetal movement and the pregnancy was carried to term. He was delivered by elective caesarean section on account of previous uterine surgeries. At birth, he was well and did not require resuscitation or hospital admission. However, at 4 months of age, he was admitted to hospital and treated for respiratory tract infection. During this admission, he was also noted to be floppy. On discharge, he was referred to our clinic for further evaluation. On physical examination, he had an appropriate social smile and bright facial expression. He had tongue fasciculations. He was floppy with reduced truncal and limb tone, and severe head lag. He had muscle weakness which was more proximal than distal, and his deep tendon reflexes were absent. Chest wall and abdominal examination showed paradoxical breathing. All other examination findings were normal. A clinical diagnosis of SMA type 1 was made. His serum creatine kinase was marginally increased at 274 U/L (normal reference: 30-190 U/L). Full blood counts and thyroid function were normal. An electrocardiogram showed baseline fibrillations and sinus tachycardia (Figure 1). Molecular genetic testing by polymerase chain reaction detected a homozygous deletion of SMN1 gene consistent with a diagnosis of SMA. The parents were counselled on the condition, its prognosis, and the various options for feeding. They opted for oral feeding but were still open to further discussions on gastrostomy tube feeding. They were taught about safe feeding practices, and the infant started physiotherapy. He was seen again on review at age 6 months and 8 months and was assessed to be growing well although gross motor development was still severely delayed. At age 9 months, he was admitted again to a district hospital with a chest infection where he succumbed to the illness. His older brother from the same parents who was 3 years at the time was doing well with no features suggestive of SMA.

**Case 2:** this is a 3-month-old female born as the third child to a couple with no consanguinity. She was seen at the neurology clinic in 2019 on account of floppiness and frequent choking during feeding. Pregnancy was uneventful. Her mother felt adequate foetal kicks and delivered at term by elective caesarean section because of two previous caesarean sections. She was well at birth and weighed 3.3kg. However, the parents were worried as their second child (case 1) had died at age 9 months after diagnosis of SMA type 1, and so self-referred to the clinic when she started having frequent chocking spells during feeding. On presentation, she had good social smile and symmetrical face. She did not have myopathic facies, ophthalmoplegia or drooling of secretions. She had tongue fasciculations. The motor examination showed a floppy infant with significant head lag, reduced spontaneous muscle movement, proximal muscle weakness and absent deep tendon reflexes. She had paradoxical breathing. All other examinations were unremarkable. Molecular genetic testing showed a homozygous deletion of the SMN1 gene. Other supplemental findings included baseline fibrillation with sinus tachycardia on ECG, normal thyroid function, normal serum creatine kinase levels and normal peripheral blood counts. Initially, the parents declined to have a gastrostomy tube inserted for feeding. On review at age 5 months, she had been treated as an outpatient in a private hospital for respiratory tract infection. The parents were re-counselled and now opted for gastrostomy tube feeding. However, before the scheduled date for the procedure, they called to inform the clinic that she had passed away. Prior to her passing, she had a cold and cough but was generally well. However, she suddenly became breathless during feeding and was rushed to a private hospital where she was pronounced dead on arrival. We think she died from aspiration of feeds. She was 7 months old when she died. The parents were later called in for a counselling session by a paediatric neurologist assisted by a clinical psychologist. They have an older child who was 5-year-old and was well. Having lost two children to SMA within two years, they decided not to have any more children.

**Case 3:** this is an 8-year-old girl who was seen in our paediatric neurology clinic in 2018. There is no consanguinity between her parents. Pregnancy was uneventful and her mother reports adequate foetal kicks during the pregnancy. She was born at term and was well at birth. She gained neck control early and sat independently between 6-7 months. Her parents become alarmed when at age 18-20 months she had not walked, though her speech and social developments were advanced. She was seen at various hospitals and clinics without a definite diagnosis. From age 2 years, she underwent physiotherapy for a considerable number of months. At 3 years, she could take a few steps without support, but regressed back into cruising a few weeks later. She had been treated on a few occasions for respiratory tract infections but had never been hospitalized. On presentation at 8 years of age, she was a bright eloquent child in a wheelchair accompanied by both parents. Her vision, extra-ocular movements and facial expressions were normal. She had tongue fasciculation, but no sign of bulbar dysfunction. Her motor examination showed coarse hand tremors, decreased muscle tone and contractures in the Achilles tendons. She also had significant muscle weakness which were more proximal than distal. Her deep tendon reflexes were absent. She had a thoracolumbar scoliosis, convex to the left. An ECG showed a baseline fibrillation and normal wave patterns. An initial impression of SMA type III was made and later confirmed when her molecular genetic testing showed homozygous deletion of SMN1.The family were counselled, and she transitioned into an electric wheelchair with solid back seating. The family also made some architectural changes in their home and this has significantly improved her mobility. She has been seen regularly on review at the clinic and at physiotherapy. Her forced vital capacity has remained above 50% and the scoliosis has not worsened. She attends mainstream school. Her school which is a two-storey block decided to keep her class on the ground floor until she completes her junior high grades. Currently at age 11 years, she is doing well in school.

**Figure 2:**
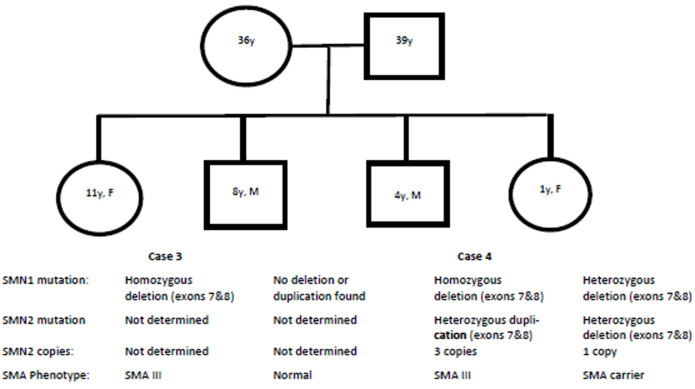
family pedigree of cases 3 and 4 showing the results of molecular genetic testing and the phenotype of all four children

**Case 4:** this is a 4-year-old boy, and a younger sibling of case 3. She is the third of four siblings all from the same parents. At the time his sister was diagnosed with SMA type III, he was a baby with mild delay in motor development. At age 12 months, he was bottom shuffling and could not pull to stand. He walked independently at age 18 months. Currently at age 4 years, he has a waddling gait and a Gower´s sign. He has an advanced social and speech development. His motor examination showed proximal muscle weakness and areflexia. He is generally well with no history of hospitalization. He has no abdominal breathing. His respiratory function has been monitored with FVC consistently above 50%. Following the genetic counselling after his sister was diagnosed with SMA type III, their parents decided to test all their four children for SMN1 and SMN2 mutations and copy numbers. He tested homozygous recessive for SMN1 deletion on exons 7 and 8 and had a heterozygous duplication of SMN2 on exons 7 and 8 with 3 copy numbers of SMN2. The second child of this family is an 8-year-old female with no deletion of the SMN1 gene. The fourth child is a carrier with a heterozygous deletion of both SMN1 and SMN2 genes. The family tree is shown in Figure 2.

**Case 5:** a 9-month-old girl born in the United States to non-consanguineous Ghanaian parents. The pregnancy was carried to term. The mother, though her first pregnancy, thinks the foetal kicks were adequate. She was well at birth but was diagnosed with SMA through newborn screening in the United States. She was offered genetic therapy with onasemnogene abeparvovec-xioi but the parents refused the treatment until she was at age 4 months. At 9 months of age, the family relocated to Ghana and was referred to our clinic for continuity of care. On examination, she had a bright facial expression and normal extraocular movements. There were no tongue fasciculations, but she pooled her oral secretions. She had a significant head lag and decreased truncal and limb tones with areflexia. Her ECG showed baseline fibrillations. Following the initial genetic counselling, the mother requested to have a molecular genetic test for herself to confirm her carrier status. The test revealed she had a heterozygous deletion for the SMN1 gene at exon 7 with 1 copy number of the SMN2 gene indicating that she is an SMA carrier. The patient was admitted to hospital at age 16 months for respiratory tract infection and received antibiotics and chest physiotherapy. Currently at 18 months old, her speech and social developments are well advanced. She has many words in her vocabulary and started to use 2-3 word sentences and maintains good social communication. However, she has significant motor delay. At 18 months, she has attained neck control but can only sit with support. The family has acquired a suctioning machine for use at home and plans to acquire a BiPAP machine for nocturnal non-invasive ventilation. We have also started discussions on placing a gastrostomy tube for feeding.

## Discussion

Spinal muscular atrophy is a motor neuron disease which results from a deletion in the survival motor neuron 1 (SMN1) gene and inherited as an autosomal recessive disorder. Classification of SMA, though based on clinical criteria, has a correlation with SMN2 copy number. More copies of SMN2 generally imply a milder disease phenotype, though overlap prevents the use of SMN2 copy numbers as the sole predictor of disease subtype and severity [1]. In Ghana, there has been only a few reported cases of SMA in the literature. It is likely that most children with SMA are missed or misdiagnosed. Case 3 in this study was initially misdiagnosed with various conditions including cerebral palsy until she was evaluated at a tertiary pediatric neurology clinic. The factors contributing to the initial misdiagnosis and late diagnosis include the lack of expertise or clinicians with the requisite knowledge and experience. There are only a few pediatric neurologists and neuro-disability specialists for a population of 30 million people. Also, molecular genetic testing is not available in the public health system. The cases presented in this study had their genetic testing done in private laboratories outside the country. This makes the testing expensive and not within the reach of the average Ghanaian. Other investigations such as muscle biopsy are considered too invasive and are currently not favored in most neuromuscular centers. Neurophysiological examinations such as electromyogram and nerve conduction studies are not available, and where available are usually not affordable to many. Newborn screening programs have become a key avenue for early diagnosis of children with SMA in advanced countries. These programs are not available in low- and middle-income countries like Ghana, contributing to the missed and late diagnosis in cases of SMA. Of the five cases presented, only one was picked early from a newborn screening program in the United States. A key step in the diagnosis of four of our five cases was the careful history and the detailed physical examination performed at the clinic. In the absence of muscle biopsy, genetic and neurophysiological testing, a careful history and detailed neurological examination remain priceless to the neurologist or pediatrician in resource-limited settings like ours. Sometimes, it is important to go over the history and physical examination twice or more to be able to pick important clues for diagnosis. A relatively simple test to perform is the ECG. In children with SMA, the ECG shows tremor of the baseline. This was seen in 4 of our cases who had an ECG done. Serum creatine kinase assay is important as it helps to exclude a diagnosis of muscular dystrophy [1].

The management of SMA in resource-limited settings is equally challenging. First, cases may present late with severe neuro-disabilities and the inability to perform molecular genetic testing means that often there is no diagnostic closure. In the Komfo Anokye Teaching Hospital, as in most other tertiary hospitals in Ghana, there are no geneticists or genetic counsellors. As such, clinicians must take on the duty of genetic counselling for families with SMA. In cases 1 and 2, we were successful in our counselling approach as the parents of the children came to understand the genetics of the disorder and the implications for future pregnancies and took the informed decision not to have any more children, but perhaps a geneticist or a genetic counsellor could have done this better. There are no dedicated neuromuscular clinics in the country. At KATH, we recently started a multidisciplinary neuro-metabolic and neuro-disability clinic where such cases can be followed. Bringing onboard a pulmonologist will improve the monitoring of respiratory function and enhance the quality of life of these patients and many others. Genetic modification therapies have recently been approved by the US Food and Drug Administration and other regulatory bodies in Europe [8]. These treatments are not available in Ghana and our management is purely supportive. The only one of our cases who received genetic therapy had the treatment done in the United States. Also, respiratory care in steady states and during intercurrent illness is a challenge in limited-resource settings like ours. Only one out of our 3 cases with SMA I could afford a suctioning machine at home and nocturnal noninvasive respiration. These challenges are complicated by cultural, traditional, and religious beliefs which sometimes seem to provide a “better” explanation for neuro-disabilities like SMA. In cases 1 and 2, the parents refused gastrostomy tube insertions. This has been the case in many other patients with neurological disorders in which such interventions are discussed with the parents. It usually takes a lot of counselling sessions before they accept to have these procedures. Many a time, there is the notion that these conditions have spiritual undertones and cannot be managed in hospital. These notions are usually fueled by traditional and religious practitioners.

## Conclusion

Cases of SMA in Ghanaian children are not widely reported due to challenges with the diagnosis. The best approach remains a detailed history and physical examination. Confirmation by molecular genetic testing is only recently being done in private laboratories who send samples overseas to be tested, making it unaffordable for many. Supportive tests such as neurophysiology investigations and muscle biopsies are not readily available. Also, there is no new-born screening programme for SMA in Ghana. The management is equally challenging due to the lack of expertise in many parts of the country. The future of SMA care in Ghana will depend on improving expertise, access to molecular genetic testing and other supplemental investigations as well as genetic modification therapy. New-born screening programmes will aid in early diagnosis

### What is known about this topic

- *Spinal muscular atrophy is caused by homozygous deletion in SMN 1 gene, while the severity of the disease is determined by the copy numbers of SMN 2 gene*;
- *Most patients with SMA type 1 die from respiratory failure due to aspirations in the first year of life*;
- *Management of patients with SMA in resource-limited settings is mainly supportive and includes a multi-disciplinary team approach*.

### What this study adds

- *In resource-limited settings, cases of SMA may be misdiagnosed due to lack of expertise and non-availability of diagnostic tests*;
- *Careful histories and detailed physical examinations are key in making the diagnosis of SMA, especially in resource-limited settings*.
